# Limited Predictive Performance of the SAMe-TT₂R₂ Score for Suboptimal Time in Therapeutic Range (TTR) in Thai Patients With Atrial Fibrillation: A Retrospective Analysis

**DOI:** 10.7759/cureus.93555

**Published:** 2025-09-30

**Authors:** Panisa Manasirisuk, Thunchanok Kuichanuan, Witsarut Manasirisuk

**Affiliations:** 1 Department of Pharmacy, Faculty of Medicine, Srinagarind Hospital, Khon Kaen University, Khon Kaen, THA; 2 Department of Medicine, Faculty of Medicine, Srinagarind Hospital, Khon Kaen University, Khon Kaen, THA

**Keywords:** anticoagulants, non-valvular atrial fibrillation, same-tt2r2 score, time in therapeutic range, warfarin

## Abstract

Background: The SAMe-TT₂R₂ score is used to predict poor anticoagulation control (time in therapeutic range (TTR)) in warfarin users. However, its performance varies across ethnicities, and its validity in the Thai population is uncertain. This study aimed to evaluate the score's predictive performance and redefine the optimal cutoff for Thai patients with atrial fibrillation (AF).

Methodology: This retrospective analysis included 140 patients with non-valvular AF on warfarin at a single University Hospital. TTR was calculated using the Rosendaal method, with suboptimal control defined as TTR <70%. The score's overall predictive performance was assessed using the area under the ROC curve (AUC).

Results: The mean of TTR was 59.89% ± 28.56%, and 83 patients (59.3%) had suboptimal TTR. The SAMe-TT₂R₂ score ≥3 demonstrated poor predictive ability for suboptimal TTR (AUC = 0.55; 95% confidence interval (CI), 0.50-0.60). At this cutoff, key diagnostic metrics included high sensitivity (95.2%) but extremely low specificity (14.0%), a modest positive predictive value (PPV) of 61.7% and a low negative predictive value (NPV) of 66.7%. Notably, in multivariable analysis, age <60 years, a risk factor in the original score, was independently associated with a lower risk of suboptimal TTR. A longer duration of warfarin use was also associated with a lower risk of suboptimal TTR.

Conclusions: The SAMe-TT₂R₂ score showed poor performance in predicting suboptimal TTR in our Thai cohort and has limited clinical utility. The score cannot reliably *rule in* or *rule out* patients at risk for poor control. Clinical decisions, such as intensive monitoring or switching to a direct oral anticoagulant (DOAC), should be guided by a patient's actual TTR rather than the SAMe-TT₂R₂ score. The single-center, retrospective design and modest sample size limit generalizability and statistical power, necessitating a further prospective trial for validation.

## Introduction

Warfarin is a cornerstone oral anticoagulant for the treatment and prevention of thromboembolic events in patients with conditions such as atrial fibrillation (AF) [1]. However, its management is complex due to a narrow therapeutic index and numerous interactions, necessitating careful monitoring. The quality of anticoagulation control is measured by the time in therapeutic range (TTR), which is the percentage of time a patient’s International Normalized Ratio (INR) remains within the target range [2], although achieving an optimal TTR remains a persistent clinical challenge [3].

The clinical importance of maintaining a high TTR is well-established. A low TTR is directly associated with an increased risk of both ischemic stroke and major bleeding [4]. Recognizing this, major international guidelines, including the 2024 European Society of Cardiology (ESC) Guidelines, now consider a TTR below 70% as inadequate control. This inadequacy prompts a re-evaluation of treatment and a potential switch to a direct oral anticoagulant (DOAC) [5]. This highlights the critical need for tools that can prospectively identify patients who are unlikely to achieve an optimal TTR on warfarin.

In Thailand, warfarin was commonly prescribed to patients with AF. However, inadequate TTR has been observed in a previous report, which showed a TTR of 32.91% [6]. This result was similar to the previous data, showing that Asian countries showed a lower proportion of TTR compared to other regions of the world (31.1% vs 54.1%) [7].

To address this need, the SAMe-TT₂R₂ score was developed as a simple clinical prediction tool to help clinicians identify patients with AF at risk for poor anticoagulation control [8]. The score incorporates six factors: sex (female), age (<60 years), medical history (≥2 comorbidities), treatment (interacting drugs like amiodarone), tobacco use, and race (non-Caucasian). The original validation by Apostolakis et al. proposed that a score of ≥2 predicts a high likelihood of poor TTR [9], guiding clinicians to consider more intensive monitoring or alternative anticoagulants from the outset.

However, the SAMe-TT₂R₂ score's utility is limited in Asian populations. The *Race* component automatically assigns two points, classifying all non-Caucasian patients as high-risk (score ≥2) by default. This contradicts clinical experience, where many patients achieve good TTR, thus questioning the validity of the standard cut-off in this demographic. Beyond the "Race" component, the differing prevalence of comorbidities, lifestyle factors, and genetic polymorphisms influencing warfarin metabolism (e.g., *CYP2C9*, *VKORC1* variants) [10]. While some studies have evaluated SAMe-TT₂R₂ in other Asian groups [11,12], data specifically from Thailand have been inconsistent, and its clinical utility remains debated. These earlier studies varied in their patient populations and methodologies, leaving an ongoing need for further validation in different Thai clinical settings. This retrospective study aims to contribute to the existing body of local evidence by evaluating the predictive performance of the SAMe-TT₂R₂ score for suboptimal TTR (<70%) in a contemporary cohort of patients with AF from a single university hospital. Our objective was to rigorously assess the score's discriminatory ability and determine if any threshold offers clinical utility for guiding anticoagulant therapy in routine Thai practice.

## Materials and methods

A retrospective cohort study was conducted by analyzing data from the Warfarin Clinic of the Cardiovascular Unit at Srinagarind Hospital, a university-affiliated tertiary care center in Khon Kaen, Thailand. The study protocol received full approval from the Khon Kaen University Ethics Committee for Human Research (HE681203), and the requirement for individual informed consent was waived due to the study's retrospective design and the use of de-identified patient data. The study population was drawn from patient records between January 1, 2024, and December 31, 2024.

The inclusion criteria required patients to be adults (aged ≥18 years) with an established diagnosis of non-valvular atrial fibrillation. Patients were selected if they had at least three International Normalized Ratio (INR) measurements recorded within one year of warfarin therapy. This threshold for INR measurements was chosen to align with the typical warfarin clinic follow-up protocol of approximately three to four times per year, ensuring that included patients had consistent and clinically representative monitoring frequency within a relevant timeframe. Patients were excluded if they had fewer than three INR measurements during the observation period or had incomplete data necessary to calculate the full SAMe-TT₂R₂ score. We acknowledge that the exclusion of patients with infrequent INR monitoring could potentially bias the cohort towards more adherent patients with better follow-up records.

Data for eligible patients were extracted from the Warfarin Clinic’s pharmaceutical care program database and the main hospital information system (HIS). To ensure data accuracy and minimize extraction errors, the initial data abstraction was performed by a research assistant. Subsequently, a second independent researcher verified a 50% random sample of these extracted records, with any discrepancies resolved through discussion and consensus. The collected variables included baseline characteristics (gender, age, health insurance scheme, educational level), clinical information (primary diagnosis, comorbidities, concomitant medications), and all available laboratory values (INR, Prothrombin Time (PT)). The SAMe-TT₂R₂ score was calculated for each individual based on the original methodology described by Apostolakis et al. [9]. For the *Treatment *component, interacting drugs were defined as amiodarone, consistent with the original score derivation.

The primary outcome for this study was poor anticoagulation control. This was determined by first calculating the TTR for each patient using the standard Rosendaal linear interpolation method, with a target INR range of 2.0 to 3.0. To accurately reflect maintenance therapy, the initial 30-day warfarin induction period was excluded from all calculations, as this phase is characterized by intentional INR variability. INR testing frequency largely followed the established clinical protocols of our Warfarin Clinic, typically ranging from monthly for stable patients to more frequent during initiation or periods of instability. Poor anticoagulation control was subsequently defined as a TTR below 70%.

All statistical analyses were performed using SPSS Statistics for Windows, Version 28.0 (IBM Corp., Armonk, NY), with a two-sided *P*-value of <0.05 considered statistically significant. Descriptive statistics were used to summarize baseline characteristics, and group comparisons between patients with poor and good TTR were made using the independent t-test or Mann-Whitney U test for continuous data and the Pearson’s chi-square or Fisher’s exact test for categorical data. The predictive performance of the SAMe-TT₂R₂ score was assessed using receiver operating characteristic (ROC) curve analysis. The area under the ROC curve (AUC) with its 95% confidence interval (CI) was calculated to quantify overall accuracy. The sensitivity, specificity, positive and negative predictive values (positive predictive value (PPV), negative predictive value (NPV)), and positive and negative likelihood ratios (LR+ and LR-) were calculated. To identify factors associated with suboptimal TTR (TTR <70%), we first performed a univariable logistic regression analysis for each baseline characteristic and each component of the SAMe-TT₂R₂ score. Subsequently, a multivariable logistic regression analysis was conducted to identify independent predictors of suboptimal TTR. Results are presented as odds ratios (OR) with 95% CIs.

As this was a retrospective study utilizing existing clinical data, no formal a priori sample size calculation or power analysis was conducted. All reported analyses reflect a complete case approach for available data; no imputation strategies were employed for missing values beyond the explicit exclusion criteria.

## Results

A total of 140 patients met the inclusion criteria and were included in the final analysis. Baseline clinical and demographic characteristics are presented in Table 1. The mean age of the cohort was 67.4 ± 15.1 years, and the mean TTR was 59.9% ± 28.6%. The mean SAMe-TT₂R₂ and CHA₂DS₂-VASc scores for the entire cohort were 3.6 ± 0.8 and 1.9 ± 1.3, respectively. Overall, 83 patients (59.3%) were classified as having poor anticoagulation control (TTR < 70%).

**Table 1:** Baseline characteristics of patients. Data are presented as mean (SD) or *n* (%). CI, confidence interval; SD, standard deviation; TIA, transient ischemic attack; TTR, time in therapeutic range.

Demographic data	Total (*n* = 140)	TTR < 70% (*n* = 83)	TTR ≥ 70% (*n* = 57)	*P*-value
Female, *n* (%)	71 (50.7)	39 (47.0)	32 (56.1)	0.231
Age, mean (SD)	67.44 (15.14)	68.38 (14.69)	66.04 (15.83)	0.371
< 60 years-old, *n* (%)	35 (25.0)	16 (19.3)	19 (33.3)	0.046
Smoking, *n* (%)	0 (0)	0 (0)	0 (0)	-
Education level, *n* (%)				
Lower than secondary school	87 (62.1)	53 (63.9)	34 (59.6)	0.063
Secondary school/High school	24 (17.1)	17 (20.5)	7 (12.3)	
Bachelor's degree	22 (15.7)	13 (15.7)	9 (15.8)	
Master's degree or higher	7 (5.0)	1 (1.2)	6 (10.5)	
Health Insurance Scheme, *n* (%)				
Universal Coverage Scheme	60 (42.9)	40 (47.0)	20 (36.8)	0.472
Social Security Scheme	15 (10.7)	7 (8.4)	8 (14.0)	
Civil Servant Medical Benefit Scheme	63 (45.0)	36 (43.4)	27 (47.4)	
Self-pay	2 (1.4)	1 (1.2)	1 (1.8)	
TTR (%), mean (SD)	59.89 (28.56)	40.04 (17.73)	88.82 (11.35)	<0.001
SAMe-TT_2_R_2 _score, mean (SD)	3.59 (0.84)	3.67 (0.78)	3.48 (0.91)	0.203
CHA_2_DS_2_-Va score, mean (SD)	1.9 (1.31)	1.95 (1.29)	1.82 (1.35)	0.564
Duration of warfarin treatment (years),mean (SD)	3.19 (2.61)	2.61 (2.33)	4.05 (2.77)	0.001
Comorbidities, *n* (%)				
Hypertension	52 (37.1)	28 (33.7)	24 (42.1)	0.287
Diabetes mellitus	24 (17.1)	14 (16.9)	10 (17.5)	0.855
Stroke/TIA	14 (10.0)	9 (10.8)	5 (8.8)	0.783
Heart failure	13 (9.3)	8 (9.6)	5 (8.8)	1.00
Coronary artery disease	2 (1.4)	2 (2.4)	0 (0)	0.517
Peripheral arterial disease	11 (7.9)	10 (12.0)	1 (1.8)	0.029
Hepatic disease	4 (2.9)	4 (4.8)	0 (0)	0.098
Chronic kidney disease	9 (6.4)	6 (7.2)	3 (5.3)	0.673
Pulmonary disease	0 (0)	0 (0)	0 (0)	-
Polypharmacy, *n* (%)	62 (44.3)	38 (45.8)	24 (42.1)	0.863
Drug interaction, *n* (%)	23 (16.4)	17 (20.5)	6 (10.5)	0.166

Univariable and multivariable logistic regression analyses were performed to identify predictors of poor TTR (Table 2). In the multivariable model, a longer duration of warfarin treatment was independently associated with a lower likelihood of poor TTR (adjusted OR (aOR) 0.86, 95% CI 0.74-0.99; p=0.038). Notably, age <60 years was also independently associated with a significantly lower risk of poor TTR (aOR 0.35, 95% CI 0.15-0.84; *P *= 0.014).

**Table 2:** Univariate and multivariate analyses of factors associated with suboptimal TTR (TTR < 70%). TTR, time in therapeutic range

	Univariable analysis		Multivariable analysis	
Variables	Crude OR (95% CI)	*P*-value	Adjusted OR (95% CI)	*P*-value
Age <60 years	0.41 (0.19-0.89)	0.024	0.35 (0.15-0.84)	0.014
SAMe-TT_2_R_2 _score	1.34 (0.89-2.02)	0.165	1.15 (0.85-1.56)	0.367
Duration of warfarin treatment	0.81 (0.71-0.93)	0.003	0.86 (0.74-0.99)	0.038
Peripheral arterial disease	7.7 (0.95-61.71)	0.055	5.12 (0.91-28.77)	0.07

**Figure 1:**
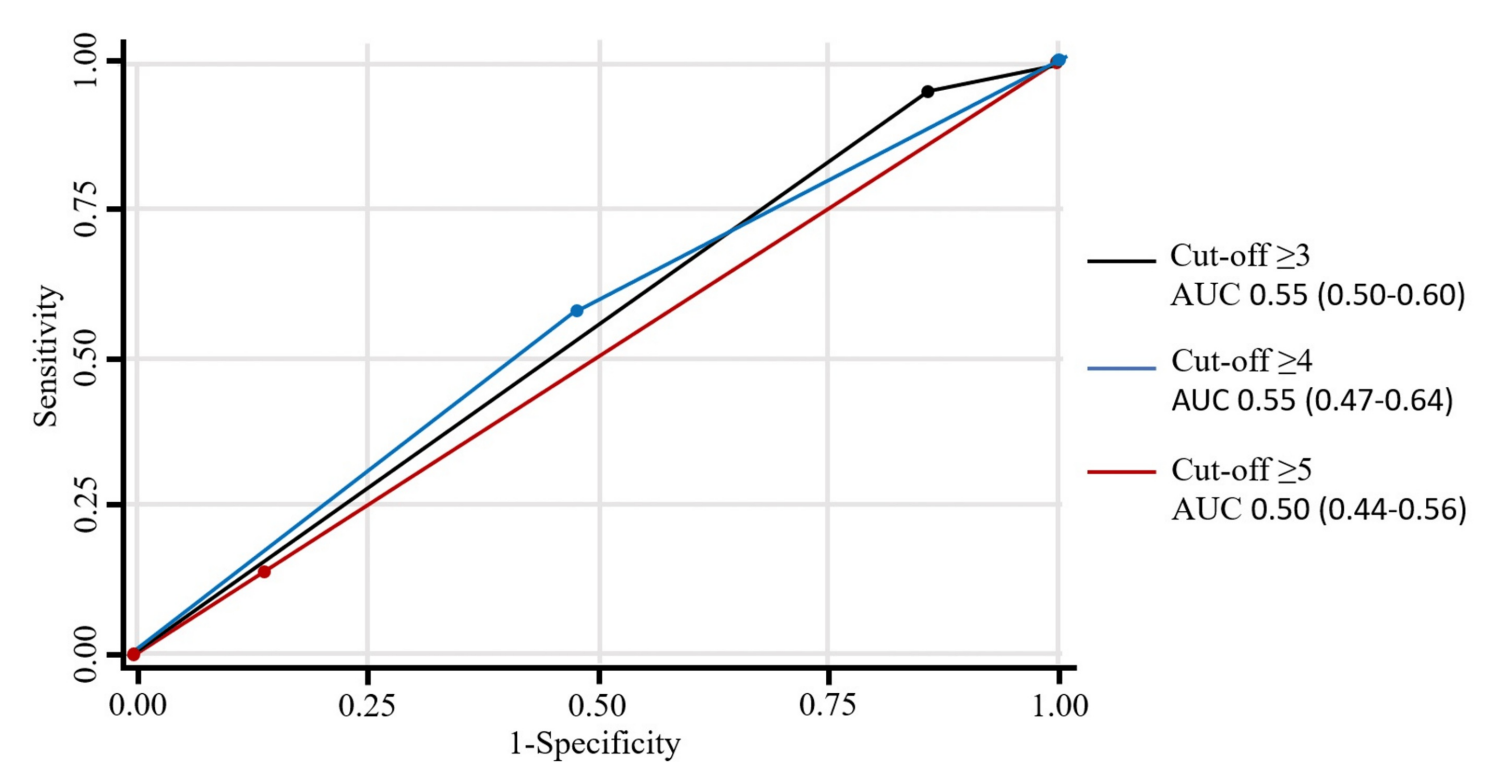
Receiver operating characteristic (ROC) curve for the SAMe-TT₂R₂ score in predicting suboptimal time in therapeutic range (TTR). AUC, area under the ROC curve

A crucial characteristic of our cohort was its non-Caucasian composition, which meant that all patients automatically received 2 points for the *Race* component of the score. Consequently, the minimum possible SAMe-TT₂R₂ score was 2, and an analysis of cutoff points <2 was not feasible. For a specific clinical application, the SAMe-TT₂R₂ score of ≥3 was identified through ROC analysis as a potential cutoff point. At this threshold, the score demonstrated an AUC of 0.55 (95% CI, 0.50-0.60), as visually represented in Figure 1. A high sensitivity of 95.2% (95% CI, 88.1-98.7), but a very low specificity of 14.0% (95% CI, 6.3-25.8). Its PPV was 61.7% (95% CI, 52.7-70.2), and its NPV was 66.7% (95% CI, 34.9-90.1). A score of ≥4 was also evaluated as an alternative cutoff point, yielding a sensitivity of 57.8% (95% CI, 46.5-68.6), a specificity of 52.6% (95% CI, 39.0-66.0), a PPV of 64.0% (95% CI, 52.1-74.8), and an NPV of 46.2% (95% CI, 33.7-59.0). While both cutoffs had comparable overall AUCs, the score ≥3 threshold was favored for its markedly higher sensitivity and NPV, making it more suitable for a broad screening approach. The detailed diagnostic metrics for all potential score cutoffs, including likelihood ratios, are shown in Table 3.

**Table 3:** The SAMe-TT2R2 score for the prediction of suboptimal TTR. AUC, area under the receiver operating characteristic curve; CI, confidence interval; NPV, negative predictive value; LR-, negative likelihood ratio; PPV, positive predictive value; LR+, positive likelihood ratio; TTR, time in therapeutic range.

Cutoff score	Sensitivity (%) (95% CI)	Specificity (%) (95% CI)	PPV (%) (95% CI)	NPV (%) (95% CI)	LR+ (95% CI)	LR- (95% CI)	AUC
≥3	95.2 (88.1-98.7)	14.0 (6.3-25.8)	61.7 (52.7-70.2)	66.7 (34.9-90.1)	1.11 (0.99-1.24)	0.34 (0.11-1.09)	0.55 (0.50-0.60)
≥4	57.8 (46.5-68.6)	52.6 (39.0-66.0)	64.0 (52.1-74.8)	46.2 (33.7-59.0)	1.22 (0.88-1.70)	0.80 (0.56-1.14)	0.55 (0.47-0.64)
≥5	14.5 (7.7-23.9)	86 (74.2 – 93.7)	60 (36.1-80.9)	40.8 (32-50.2)	1.03 (0.45-2.36)	1.00 (0.87-1.14)	0.50 (0.44-0.56)

## Discussion

In this analysis, the SAMe-TT₂R₂ score had limited ability to predict suboptimal TTR among Thai patients with AF, with an AUC of 0.55. This poor performance contrasts sharply with the original validation studies in Western populations and suggests the score is less generalizable than even previous Thai studies had reported. For instance, Leedumrongwattanakul [13] observed an AUC of 0.63, and Krittayaphong et al. [14] reported a C-statistic of 0.54. Our low AUC confirms these findings, indicating poor discriminative ability. This aligns with broader systematic reviews. A meta-analysis by Poonchuay et al. [15] noted modest accuracy across all cutoffs and concluded the score’s ability to identify patients with poor TTR remained limited, even though sensitivity was high (83.05%) at a cutoff of ≥3 in Asian patients. Similarly, another meta-analysis by Incomenoy et al. [16] found that while high scores were linked to adverse events, the score had ‘limited and inconclusive predictability for poor TTR.’ Further evidence from Methavigul [17] also showed no real difference in labile INR between high- and low-scoring patients (*P *= 0.056). Taken together, this body of evidence shows the SAMe-TT₂R₂ score lacks generalizability for predicting TTR in Thai patients.

Despite the score's limited overall performance, we examined specific cutoffs to see if any clinical value remained. A score of ≥3 had a high sensitivity of 95.2%. This could make it a useful broad screening tool. However, the major drawback is its very low specificity of 14.0%. As a result, 86% of patients with good TTR would be incorrectly flagged as high-risk. While the PPV was 61.7%, a large number of patients identified as high-risk would still achieve optimal TTR, making it an unreliable tool for "ruling in" risk. The NPV of 66.7% is also too low to confidently *rule out* poor control.

The practical consequences of the SAMe-TT₂R₂ score's inadequate predictive utility are underscored by existing clinical evidence. For instance, a randomized trial by Phrommintikul et al. [18] in a Thai population failed to demonstrate an improvement in TTR after implementing an educational intervention for patients with a SAMe-TT₂R₂ score ≥3. This critical result shows that even when used to guide therapy, the score does not identify patients who will benefit from standard educational strategies. It simply cannot functionally separate patients who need different interventions. A patient with a low score cannot be considered low-risk, because many factors the score ignores, like adherence, diet, other medications, and health literacy, have a major impact on TTR [19-21]. All warfarin users, regardless of their score, require comprehensive care. Therefore, decisions about intensive monitoring or switching to DOACs should be based on a patient's actual TTR, not the SAMe-TT₂R₂ score.

Our findings starkly contrast with the original validation by Apostolakis et al. [9], which reported a c-index of 0.70-0.72. The difference is likely the patient population; the original study was conducted mainly in Caucasian and African American patients. The score's 'Race' component acts as a proxy for genetic factors like *CYP2C9 *and *VKORC1* polymorphisms, which affect warfarin metabolism and vary across ethnicities [10]. Indeed, Liu et al. [22] showed that substituting the 'Race' category with genotype data significantly improved the score’s accuracy in an Asian population. While promising, genotype-guided dosing is not yet practical in Thailand due to cost, limited lab access, and a lack of local data proving its value. Therefore, although the genetic basis for these differences is clear, a genetic approach is not yet a routine clinical option. Furthermore, our study identified a notable contradiction regarding the 'Age' component. Whereas the original SAMe-TT₂R₂ score assigns a risk point for age <60 years, our multivariable analysis found that this age group was significantly associated with good TTR. One potential explanation for this inverse finding may be the higher prevalence of polypharmacy observed among older patients in our cohort, which could increase the risk of drug-drug interactions and consequently lead to greater INR instability.

The interpretation of our findings is subject to certain limitations. First, the single-center, retrospective design may limit the generalizability of our findings. Second, our modest sample size contributed to a wide confidence interval for the AUC, reflecting statistical imprecision. Third, we could not account for all potential confounding factors known to affect TTR, such as the patient's cognitive function, health literacy, or specific type of AF (e.g., paroxysmal vs. persistent), which have been shown to influence anticoagulation control [20,21]. We also acknowledge that socioeconomic factors like education and insurance schemes, while not statistically significant here, may play a crucial role and warrant investigation in larger studies.

Looking forward, rather than continuing to validate a poorly fitting score, future research should focus on developing or recalibrating a new risk score specifically for Thai and Southeast Asian populations. A prospective, multicenter study would be ideal to collect high-quality data on a wider range of potential predictors, including genetic markers, if feasible, socioeconomic factors, and patient-reported outcomes. Furthermore, investigating whether combining a modified SAMe-TT₂R₂ score with other tools, such as a formal adherence assessment, could enhance its predictive power is another promising avenue for future research.

## Conclusions

The SAMe-TT₂R₂ score demonstrated poor predictive performance for suboptimal TTR in our Thai cohort, reflected by a low AUC, and thus has limited clinical utility. While a cutoff of ≥3 had high sensitivity, its low NPV (66.7%) prevents its use as a *rule-out* tool, and its extremely low specificity (14.0%) makes it ineffective for "ruling in" patients at high risk. Our findings, constrained by a retrospective, modest sample size and single-center design, indicated that the SAMe-TT₂R₂ score was not a useful instrument for stratifying warfarin management in Thai patients. Clinical decisions, such as intensive monitoring or a switch to a DOAC, should be guided by a patient's actual TTR and individual factors, also considering the cost and accessibility of DOACs in Thailand. A large, prospective, and multicenter trial is warranted to confirm these findings. Future research should prioritize the development of a new predictive model that incorporates more relevant predictors for this population.
